# Estimating sensitivity of the Kato-Katz technique for the diagnosis of *Schistosoma mansoni* and hookworm in relation to infection intensity

**DOI:** 10.1371/journal.pntd.0005953

**Published:** 2017-10-04

**Authors:** Oliver Bärenbold, Giovanna Raso, Jean T. Coulibaly, Eliézer K. N’Goran, Jürg Utzinger, Penelope Vounatsou

**Affiliations:** 1 Epidemiology and Public Health, Swiss Tropical and Public Health Institute, Basel, Switzerland; 2 Faculty of Science, University of Basel, Basel, Switzerland; 3 Centre Suisse de Recherches Scientifiques en Côte d’Ivoire, Abidjan, Côte d’Ivoire; 4 Unité de Formation et de Recherche Biosciences, Université Félix Houphouët-Boigny, Abidjan, Côte d’Ivoire; RTI International, UNITED STATES

## Abstract

The Kato-Katz technique is the most widely used diagnostic method in epidemiologic surveys and drug efficacy trials pertaining to intestinal schistosomiasis and soil-transmitted helminthiasis. However, the sensitivity of the technique is low, particularly for the detection of light-intensity helminth infections. Examination of multiple stool samples reduces the diagnostic error; yet, most studies rely on a single Kato-Katz thick smear, thus underestimating infection prevalence. We present a model which estimates the sensitivity of the Kato-Katz technique in *Schistosoma mansoni* and hookworm, as a function of infection intensity for repeated stool sampling and provide estimates of the age-dependent ‘true’ prevalence. We find that the sensitivity for *S. mansoni* diagnosis is dominated by missed light infections, which have a low probability to be diagnosed correctly even through repeated sampling. The overall sensitivity strongly depends on the mean infection intensity. In particular at an intensity of 100 eggs per gram of stool (EPG), we estimate a sensitivity of 50% and 80% for one and two samples, respectively. At an infection intensity of 300 EPG, we estimate a sensitivity of 62% for one sample and 90% for two samples. The sensitivity for hookworm diagnosis is dominated by day-to-day variation with typical values for one, two, three, and four samples equal to 50%, 75%, 85%, and 95%, respectively, while it is only weakly dependent on the mean infection intensity in the population. We recommend taking at least two samples and estimate the ‘true’ prevalence of *S. mansoni* considering the dependence of the sensitivity on the mean infection intensity and the ‘true’ hookworm prevalence by taking into account the sensitivity given in the current study.

## Author summary

The World Health Organization (WHO) has defined a roadmap for schistosomiasis and soil-transmitted helminthiasis morbidity control and interruption of transmission with targets to be reached by 2025. Control efforts require reliable estimates of at-risk populations, number of infections, and disease burden estimates in population subgroups in terms of age and location. Intervention guidelines are based on insensitive diagnostic techniques, such as the Kato-Katz method and do not take into account the effect of sampling effort and infection intensity. Our proposed methodology estimates the infection intensity-dependent sensitivity and the ‘true’ age-prevalence of the blood fluke *Schistosoma mansoni* and hookworm. We also provide recommendations on the number of stool samples required and the methodology to be used to reliably estimate the ‘true’ prevalence of parasitic worm infections.

## Introduction

Soil-transmitted helminthiasis (STH) and schistosomiasis are two of the most prevalent neglected tropical diseases with more than 1 billion and over 250 million people affected worldwide, respectively [1, 2]. Their collective global burden is 6 million disability-adjusted life years [3], with school-aged children at the highest risk of associated morbidity. Control efforts have intensified over the past 15 years, with preventive chemotherapy serving as the main pillar [4–6].

Information about the spatial and temporal distribution of STH and schistosomiasis are important to guide interventions. Moreover, it is necessary to know which age groups contribute most to transmission, in terms of helminth egg output, in order to effectively use the available resources. There are two approaches to obtain this information. First, large-scale, standardized studies including all age groups with intense sampling which, however, is difficult to pursue due to the high cost. Second, reanalysis of data from previously published studies. Inference is hampered by the paucity of quality data [7–9]. Individual-level data are usually not reported; instead, only the number of participants tested positive for a specific helminth infection is considered. In addition, diagnosis relies on the Kato-Katz technique [10], i.e., counting of helminth eggs in a small amount of stool. This approach, however, has a low, setting-dependent sensitivity, which is governed by variation in day-to-day production of eggs per worm, non-random distribution of eggs within a stool sample, decay of eggs in the sample due to methods and duration of the experimental procedure, transportation, and storage [11–15]. Collecting multiple stool samples over consecutive days increases the accuracy but there are no guidelines on the optimal number of samples [16, 17]. Consequently, the comparison of studies that employed different sampling efforts, which is necessary for monitoring progress of control programs, is hampered.

Statistical modeling can help studying the age-prevalence and its dependence on the diagnostic error but has been restricted by the aforementioned limitations that compromise the quality of the data [7, 18]. Although the qualitative shape of helminthiasis age-prevalence curves is known, there has been little progress in the application of quantitative transmission models, especially for STH infections [19–22]. Furthermore, the dependency of the intensity of the infection on the diagnostic sensitivity has been largely neglected. The negative binomial distribution has commonly been used to fit helminth egg count data. For example, De Vlas and Gryseels [12] and Levecke et al. [23] proposed models that separate the measurement process from the true underlying infection intensity distribution. However, none of the preceding models are able to infer on the dependence of the sensitivity of the Kato-Katz method for repeated stool sampling on infection intensity.

We developed a model for fecal egg-count (FEC) data, which quantifies the relation between sampling effort, infection intensity, and diagnostic sensitivity. The model separates the infected from the non-infected individuals and the measurement process from the infection status. Variability due to egg output, experimental conditions, and aggregation within a population are taken into account. We calculate the ‘true’ prevalence and other biological and transmission-related parameters based on the probability of false-negatives. Our model improves estimation of the age-related disease burden and provides inputs for mathematical transmission models.

## Materials and methods

### Ethics statement

This study consists of a secondary analysis of published data. Ethics approval, written informed consent procedures, and treatment of infected individuals have been described elsewhere [24–27].

### Data

We tested our model performing a secondary analysis of individual level FEC data from three separate studies in medium and high transmission settings in Côte d’Ivoire conducted in 1998, 2002, and 2011, respectively. All data used were from baseline surveys with no previous mass drug administration in the area. The studies took place in Fagnampleu [24, 25], Zouatta [26], and Azaguié [27]. Based on the Kato-Katz assay, hookworm prevalence varied from 11.4% to 59.0%, and mean or infected population based infection intensity from 280 eggs per gram of stool (EPG) to 396 EPG. For *S. mansoni*, prevalence varied from 35.6% to 76.3%, and infection intensity from 152 EPG to 307 EPG. Between two and four stool samples were collected and analyzed on consecutive days of a total of 1423 participants. Azaguié and Zouatta were surveys performed in the full age range from 0 to 90 years, while Fagnampleu only included school-aged children. Prevalence of *Ascaris lumbricoides* and *Trichuris trichiura* were too low to be analyzed. Summary measures are included in Table 1, a more detailed description can be found in S1 Appendix, and the individual level data used is included in S1 Table.

**Table 1:** Resulting parameters of the simulations (mean and 95% BCI^1^) parameters of the mean infection and of the prevalence of *S. mansoni* and hookworm in Azaguié, Zouatta, and Fagnampleu in Côte d’Ivoire. Observed prevalence is the ratio of positively tested individuals in the original study, observed mean infection the arithmetic mean egg count of the individuals with a positive test.

	*S. mansoni*			Hookworm		
Parameters	Azaguié	Zouatta	Fagnampleu	Azaguié	Zouatta	Fagnampleu
	(N = 500, k = 2)^2^	(N = 559, k = 3)	(N = 354, k = 4)	(N = 500, k = 2)	(N = 559, k = 3)	(N = 354, k = 4)
Observed prevalence (%)	35.6 (31.4, 39.8)	40.8 (36.7, 44.9)	76.3 (71.8, 80.5)	11.4 (8.6, 14.2)	35.4 (31.5, 39.4)	59.0 (53.9, 64.1)
Estimated ‘true’ prevalence (%)	49.3 (40.4, 61.2)	59.6 (50.7, 69.3)	83.8 (78.3, 89.3)	14.3 (10.9, 18.5)	43.7 (38.6, 49.2)	62.2 (56.6, 67.6)
Observed mean infection (EPG)^3^	179 (171, 188)	152 (141, 163)	307 (289, 325)	396 (326, 466)	331 (301, 361)	283 (260, 306)
Estimated mean infection (EPG)^4^	132 (101, 167)	104 (84, 128)	282 (249, 321)	220 (150, 316)	261 (208, 325)	262 (221, 312)
Sensitivity^5^						
1 sample (%)	59.4 (47.6, 70.2)	48.0 (40.8, 55.8)	70.2 (66.1, 74.1)	57.1 (44.5, 68.8)	47.1 (41.7, 52.5)	53.8 (50.1, 57.7)
2 samples (%)	72.9 (59.5, 84.6)	62.3 (53.5, 71.3)	83.5 (79.3, 87.3)	81.0 (69.1, 90.1)	71.8 (65.9, 77.3)	78.5 (74.59, 81.9)
3 samples (%)	-	69.0 (59.8, 78.2)	88.2 (84.2, 91.8)	-	84.9 (80.0, 89.1)	89.9 (87.3, 92.2)
4 samples (%)	-	-	90.7 (86.8, 94.0)	-	-	95.2 (93.6, 96.6)
Day-to-day variation (*r*)	1.10 (0.80, 1.46)	0.83 (0.67, 1.02)	0.87 (0.77, 0.99)	0.25 (0.15, 0.37)	0.15 (0.13, 0.19)	0.20 (0.17, 0.23)
Aggregation (*α*)	0.09 (0.05, 0.13)	0.08 (0.05, 0.11)	0.05 (0.04, 0.07)	0.22 (0.04, 0.90)	0.32 (0.06, 1.23)	0.19 (0.05, 0.68)

^1^ Parameter posterior mean estimates and 95% Bayesian credible interval

^2^ *k* is the number of samples taken in a study; *N* the number of participants with at least one sample

^3^ The observed mean infection is the mean egg count of all individuals tested positive in at least one Kato-Katz thick smear

^4^ The estimated mean infection is *μ*_*f*_ + *μ*_*m*_

^5^ The sensitivity was calculated using eq 4

### Model

We utilized a hierarchical Bayesian model to address the objectives given in the introduction. Let *Y*_*ij*_ be the FEC, i.e., the number of helminth eggs found in sample *j* in individual *i*, *k*_*i*_ the number of stool samples from individual *i*, and *x*_*i*_ the age of individual *i*.

We assumed that a population consists of a proportion of infected individuals *p*, i.e., people that carry at least one pair of worms, and that of uninfected individuals. Thus *p* is interpreted as the prevalence. Each infected individual has a characteristic infection intensity λ_*i*_, measured in units of mean eggs per sample and assumed to be distributed within the population according to a shifted gamma distribution, given by

$$\begin{array}{c} \begin{array}{cc} \lambda_{i} & =v_{i}+\mu_{m}\\ v_{i} & ∼\text{Gamma}(\mu_{f}\cdot \alpha ,\alpha )\\ & =\frac{\alpha^{\mu_{f}\cdot \alpha }}{\Gamma (\mu_{f}\cdot \alpha )}\;v_{i}^{\mu_{f}\cdot \alpha -1}\;\exp\left(-\alpha \cdot v_{i}\right) \end{array} \end{array}$$ (1)

with a mean number of eggs *μ*_*f*_ + *μ*_*m*_ in an infected individual, variance $\frac{\mu_{f}}{\alpha }$ that corresponds to the aggregation of infection intensities, and hence, the aggregation of worms within the population. The shift parameter *μ*_*m*_ is the mean number of eggs per sample that can be expected from an individual carrying exactly one female worm and thus the minimal possible infection intensity. Direct inference on the worm load is not possible in this frame as the dependence on mean egg output is non-linear and not well known [28].

The process of taking *k*_*i*_ samples from an infected individual *i* with infection intensity λ_*i*_ is modeled by a negative binomial distribution with mean λ_*i*_ and a variance given by $\lambda_{i}+\lambda_{i}^{2}/r$. *r* reflects the additional variation, due to changes in the day-to-day helminth eggs output, the aggregation of eggs in stool, and the precise experimental procedure but not the within-population variation which is given by *α*. If a perfectly random distribution of the eggs and perfect measurement is assumed *r* → ∞, the measurement process becomes a Poisson process. By including the uninfected, the model is written as

$$\begin{array}{c} Y_{i}∼\{\begin{array}{cc} \left(1-p\right)+p\cdot \text{NB}{\left(0,\lambda_{i},r\right)}^{k_{i}}\; & \text{,}\;\text{if}\;I_{i}=0\\ p\cdot \prod_{j=1}^{k_{i}}\text{NB}\left(Y_{ij},\lambda_{i},r\right)\; & \text{,}\;\text{if}\;I_{i}=1 \end{array} \end{array}$$ (2)

which corresponds to a zero-inflated negative binomial model with *p* corresponding to the mixing proportion. *NB* is the negative binomial distribution and *I*_*i*_ the result of the Kato-Katz test over all samples from an individual. They are defined as follows:

$$\begin{array}{ll} I_{i} & =\{\begin{array}{ll} 0 & ,\;\text{if}\;\text{max}\left(Y_{i}\right)=0\\ 1 & ,\;\text{if}\;\text{max}\left(Y_{i}\right)\neq 0 \end{array}\\ \text{NB}\left(y,\lambda ,r\right) & =\left(\begin{array}{l} \begin{array}{c} y+r-1\\ y \end{array} \end{array}\right){\left(\frac{\lambda }{\lambda +r}\right)}^{y}{\left(\frac{r}{r+\lambda }\right)}^{r} \end{array}$$ (3)

False-negatives are included in the model as repeated zero measurements for an infected individual. Thus, the sensitivity depending on the number of repeated measurements becomes

$$\begin{array}{c} s_{i}\left[k_{i}\right]=1-\text{NB}{\left(0,\lambda_{i},r\right)}^{k_{i}}=1-{\left(\frac{r}{\lambda_{i}+r}\right)}^{k_{i}\cdot r} \end{array}$$ (4)

where *s* is the sensitivity, and *k* and λ vary for each individual.

Low-rank thin-plate splines are used to study the age dependence of *p*, *μ*_*f*_, *r*, and *α*. For a detailed derivation of the spline model, see Crainiceanu et al. [29]. The representation of *p* is

$$\begin{array}{c} \mathrm{logit}\left(p_{i}\right)=\beta_{0}+\beta_{1}\;x_{i}+\sum_{m=1}^{M}u_{m}\;{\left(x_{i}-\kappa_{m}\right)}^{3} \end{array}$$ (5)

where **Θ**_1_ = (*β*_0_, *β*_1_, *u*_1_, …, *u*_*M*_)^*T*^ is the vector of regression coefficients and *κ*_1_ < ⋯ < *κ*_*M*_ are the fixed knots. The other parameters can be represented analogously using logarithmic or linear spline models. The spline regression makes only a few very general assumptions about the shape of the curve, e.g., continuity and differentiability, and is therefore able to infer without requiring prior knowledge about the biology of the process, i.e., the transmission model.

The minimum eggs per sample *μ*_*m*_ is fixed to the average egg output of a worm divided by an average amount of feces per day, multiplied by the weight of a sample. For hookworm, *μ*_*m*_ is 5 eggs in a sample which corresponds to 120 EPG and for *S. mansoni*, *μ*_*m*_ is 0.03 eggs which corresponds to 0.72 EPG [30, 31]. We choose the following semi-informative priors for the model: gamma for *r* with mean 1 and variance 1; normal for log(*α*) with mean 0 and variance 1; gamma for *μ*_*f*_ with mean 2 and variance 4; normal for *β*_0,1_ with mean 0 and variance 1; normal for *u*_1,…,*M*_ with mean 0 and variance *τ*, where *τ* is distributed as a gamma with mean 2 and variance 4. The results were not sensitive to the specific shape of the priors.

Bayesian inference was performed using Markov chain Monte Carlo (MCMC) simulations implemented in Stan [32]. Validity of the model was checked using simulated data. Models with splines on *p*, *μ*_*f*_, *r*, and *α* were run to check for age dependence. *μ*_*f*_, *r*, and *α* showed no significant age dependence and were set as independent of age for the simulations presented in the results section. *μ*_*m*_ was varied from 1 egg to 6 eggs for hookworm and from 0.01 eggs to 0.1 eggs for *S. mansoni*, which also showed no significant influence. The lower limit of 0.01 eggs per slide for *S. mansoni* corresponds to roughly 100 eggs in 500 g of stool. The upper limit of 0.1 eggs per slide corresponds to 1000 eggs per 500 g of stool therefore any value larger than 0.1 is most likely unrealistic for a single worm pair. The model was run with a total of 25 chains, with 20,000 iterations each, of which 2,000 where used as warm up and adaption, for each study and for each of the two infections separately. Convergence was achieved, and assessed using Gelman + Rubin diagnostics and visual inspection of the chains [33].

## Results

We applied a Bayesian hierarchical model to FEC data from three studies carried out in Zouatta, Azaguié and Fagnampleu in Côte d’Ivoire, as described in the “Materials and methods” section. Parameter posterior mean estimates and 95% Bayesian credible interval (BCI) are summarized in Table 1.

### Estimated ‘true’ prevalence and its relation to age

**Fig 1:**
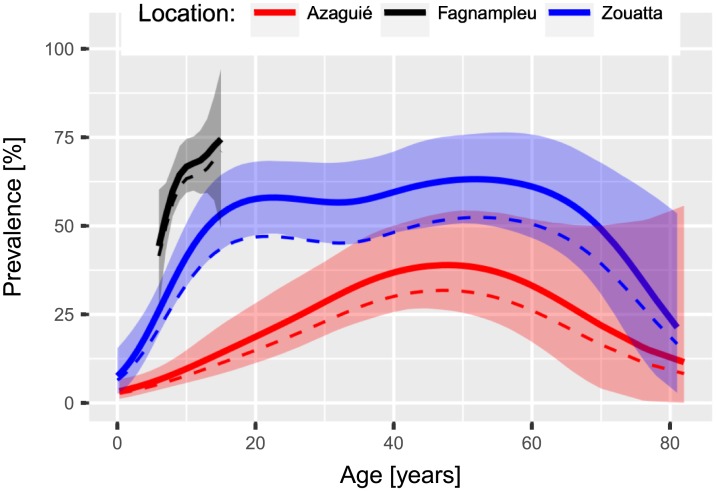
Prevalence of hookworm infection in relation to age for the three studies in Côte d’Ivoire, including 95% BCI indicated by shaded areas, and observed prevalence as dashed line.

The three studies are from different hookworm transmission settings with observed prevalence of 11.4% and mean infection intensity of an infected individual of 396 EPG for Azaguié, 35.4% and 331 EPG for Zouatta, and 59.0% and 283 EPG for Fagnampleu. Based on our model, we estimated the ‘true’ hookworm prevalence at 14.3% (95% BCI 10.9–18.5%), 43.7% (95% BCI 38.6–49.2%), and 62.2% (95% BCI 56.6–67.6%), for Azaguié, Zouatta, and Fagnampleu, respectively. The estimated mean infection intensity does not significantly differ from one study to another and mean estimates ranged from 220 EPG to 262 EPG (see Table 1). Age-prevalence curves in Fig 1 from the three studies show similar features such as a steep increase from birth till an equilibrium is reached at ages of around 20 years for Zouatta, and 45 years for Azaguié. The prevalence stays constant till an age of about 60 years from where the rate of infection declines. For Fagnampleu only the initial steep increase is visible due to the fact that no individuals older than 15 years were included.

**Fig 2:**
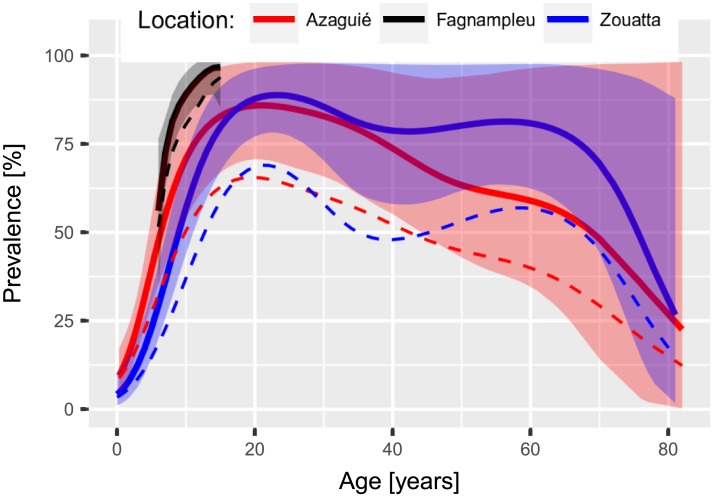
Prevalence of *S. mansoni* infection in relation to age for three studies in Côte d’Ivoire, including 95% BCI indicated by shaded areas, and observed prevalence as dashed line.

For *S. mansoni*, the Azaguié and Zouatta studies show similar transmission levels with an observed prevalence of 35.6% and 40.8% and observed mean infection intensity of 179 EPG and 152 EPG, respectively. In contrast, the study in Fagnampleu had a prevalence of 76.3% and a mean infection intensity of 307 EPG. We estimated a ‘true’ prevalence of 49.3% (95% BCI 40.4–61.2%) and a mean infection intensity of 132 EPG (95% BCI 101–167 EPG) for Azaguié, 59.6% (95% BCI 50.7–69.3%) and 104 EPG (95% BCI 84–128 EPG) for Zouatta, and 83.8% (95% BCI 78.3–89.3%) and 282 EPG (249–321 EPG) for Fagnampleu. The estimated age-prevalence curves displayed in Fig 2 show similar qualitative features. The prevalence increases up to a peak between the ages of 15 and 20 years, and subsequently declines slowly up to an age of 60 years, followed by a stronger decrease. The lower prevalence after the peak is not significant but it appears both in the Azaguié and Zouatta data.

### Variations in egg output

For hookworm, the day-to-day variation given by *r* is consistent across study sites, ranging from 0.15 (95% BCI 0.13–0.17) to 0.25 (95% BCI 0.15–0.37) (see Table 1), indicating strong overdispersion. The aggregation of egg output within the population is also consistent across the studies with *α* estimates ranging from 0.19 (95% BCI 0.05–0.68) to 0.32 (95% BCI 0.06–1.23).

For *S. mansoni* the day-to-day variation is consistent across studies and significantly different from hookworm with values ranging from 0.83 (95% BCI 0.67–1.02) to 1.10 (95% BCI 0.80–1.46). The aggregation of infections within the population shows no significant differences between studies with *α* ranging from 0.05 (95% BCI 0.04–0.07) to 0.09 (95% BCI 0.05–1.13), which indicates a significantly higher variance than for hookworm.

### Estimated sensitivity and its relation to infection intensity

For hookworm, the estimates of the diagnostic sensitivity of Kato-Katz did not vary between locations. Based on a single Kato-Katz thick smear, sensitivity estimates were in the range of 47% to 57%, for two samples obtained from different days from 72% to 81%, for three samples estimates were within the range of 85% to 90%, and for four samples around 95%. For *S. mansoni*, data from Azaguié and Zouatta revealed similar sensitivity estimates within the range of 48% to 59% for one Kato-Katz thick smear, 62% to 73% for two samples, and 69% for three samples. Fagnampleu has a higher sensitivity of 70%, 84%, 88%, and 91% for one, two, three, and four samples, respectively (see Table 1).

**Fig 3:**
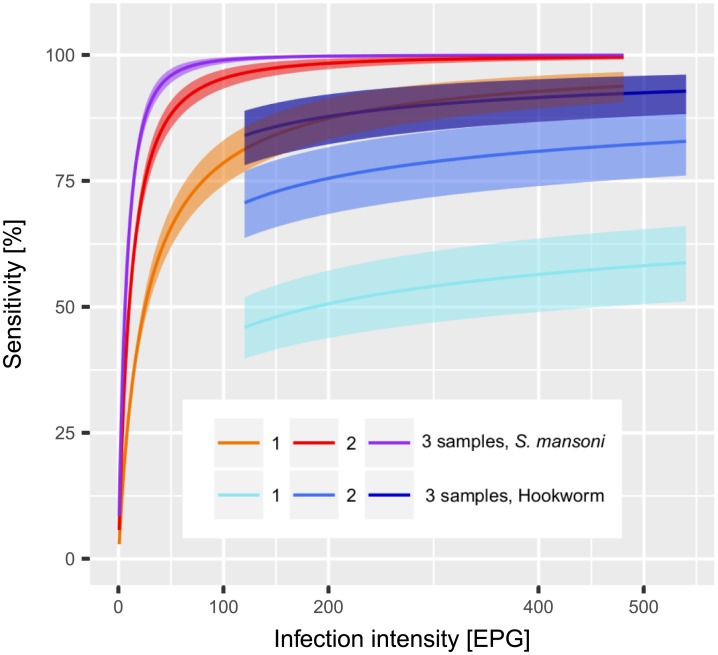
Relation between *S. mansoni* and hookworm infection intensity with Kato-Katz diagnostic sensitivity, including 95% BCI for *k* equal to 1, 2, and 3 samples per individual.

The sensitivity of the Kato-Katz technique for different infection intensities was calculated using eq 4 and is plotted in Fig 3. For hookworm the dependence on infection intensity is weak, e.g., only increasing from 40% to 55% from a very light infection of 120 EPG to a still light infection of 500 EPG. For moderate and heavy infections (>2000 EPG) the sensitivity did not significantly improve with infection intensity. However, the sensitivity can be greatly increased by examining several stool samples, e.g., for an infection intensity of 360 EPG the sensitivity can raise from 50% based on a single sample to 75% for two samples, and 92.5% for three samples.

The sensitivity was strongly associated with *S. mansoni* infection intensity. In particular, for very light infections (<5 EPG), it was below 50% even after three samples. For light infections (<100 EPG), it was still heavily dependent on infection intensity. For moderate infections (100–399 EPG), two samples gave a high sensitivity above 90%. Heavy infections (>400 EPG) were reliably detected (i.e. >99%) by testing two samples.

**Fig 4:**
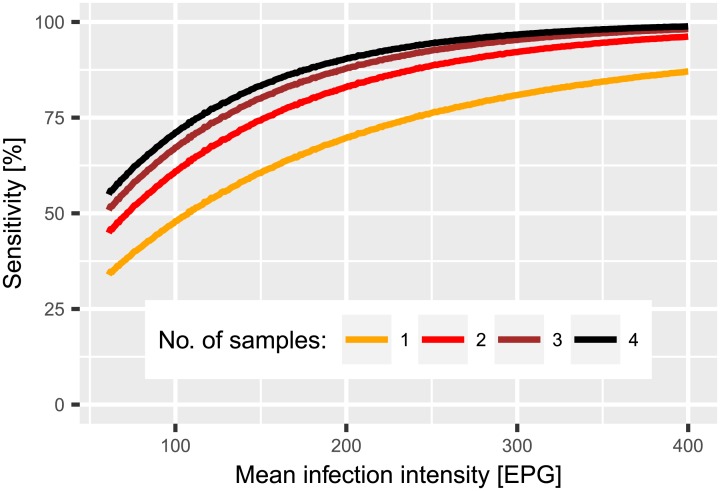
Estimated relation between sensitivity and arithmetic mean *S. mansoni* infection in a population with *α* = 0.07 and *r* = 1.0 for one to four samples.

Fig 4 shows the overall sensitivity in a population with a day-to-day variation of *r* = 1.0 and a population aggregation of *α* = 0.07 as a function of the mean infection intensity in the population. For lower transmission settings with 100 EPG comparable to Zouatta, the sensitivity after four samples is still below 75%. However, sensitivity rose to more than 95% for a setting with a mean infection intensity of over 300 EPG.

## Discussion

We present a model which determines the relation between the sensitivity of the Kato-Katz technique and intensity of *S. mansoni* and hookworm infections. The model takes into account day-to-day variations in helminth egg output and within population aggregation of worms. Additionally, we were able to test various parameters for age-dependence, especially the age structure of the prevalence.

The overall sensitivity point estimates for hookworm corroborate with estimates derived from latent class modeling approaches. Tarafder et al. [34] give an estimate of 65% sensitivity in a setting with 35% prevalence where no infection intensity is given, and Nikolay et al. [35] estimated the sensitivity for one sample at 59.5%, for two at 74.2%, and for three samples at 74.3%. For *S. mansoni* in a setting with an observed prevalence of 33%, Lamberton et al. [36] predict a sensitivity of 29.6% for one sample, 51.9% for two samples, 70.4% for three samples, and 77.8% for four samples in agreement with our estimates for Zouatta and Azaguié. The same authors predicted a much higher sensitivity of 83.5%, 97.8%, and 100% for one, two, or three samples, respectively in a setting with 95% prevalence in agreement with our estimates for Fagnampleu. Glinz et al. [37] also predicted a sensitivity of 70% for a single Kato-Katz thick smear in a high transmission setting with prevalence close to 90%, which is again consistent with our estimates for Fagnampleu.

Our estimates are in agreement with those given in the literature. However, the added value of our modeling approach is that we can predict the sensitivity depending on infection intensity and the ‘true’ prevalence in various settings and we can understand the factors influencing the sensitivity for hookworm and *S. mansoni*.

There are three main parameters that directly influence the sensitivity, i.e., the minimum infection intensity, the day-to-day variation, and population aggregation. These parameters are specific for *S. mansoni* and hookworm. The minimum infection *μ*_*m*_, which is fixed to 0.03 eggs per sample for *S. mansoni* and 5 eggs per sample for hookworm, is based on estimates of egg output for a single worm. The estimates carry a large uncertainty which were shown to be non-critical for the results. The low output of eggs per worm in *S. mansoni* makes it almost impossible to detect light infections with only a few female worms. The low sensitivity for light infections can also be seen in Fig 3 where the three *S. mansoni* curves show very low sensitivity when approaching 0 EPG. For hookworm, the output of a single female worm is about 5 eggs per sample, which already leads to a reasonable probability for detection.

The parameter *r* is determined by the day-to-day variation. The three studies agree on a value between 0.17 and 0.19 for hookworm and between 0.8 and 0.99 for *S. mansoni*. Both values conclusively contradict a Poisson process which has *r* → ∞ and could be interpreted as worms producing and excreting eggs randomly. Thus, our model clearly indicates that a worm produces eggs in a clustered fashion. A single pair of *S. mansoni* produces in the order of 100 eggs per day [30], while a female hookworm sheds around 10,000 eggs [31]. Thus, an infection that manifests itself with a similar egg count for both diseases indicates a much higher number of *S. mansoni* compared to hookworm. This difference in worm numbers has important implications, because the variation in egg output of a single worm is partly averaged out over the population of worms that produce eggs independently in an individual. The much lower variation in day-to-day output of *S. mansoni* can therefore be explained by the, on average, larger number of worms in an infected individual. The larger variation for hookworm leads to a lower sensitivity of the Kato-Katz technique for this helminth species for a similar infection intensity. Thus, the increase in sensitivity due to repeated sampling is much larger for hookworm (see Fig 3).

The population aggregation parameter *α* describes the distribution of worm output within the population. A small value indicates a relatively wider distribution, while a larger value indicates a more uniform distribution. The estimate of *α* has larger uncertainty for hookworm compared to *S. mansoni*, most likely due to the large day-to-day variation for the former species. Furthermore, *α* is overall larger for hookworm compared to *S. mansoni* indicating that egg output is more evenly distributed among individuals infected with hookworm than for *S. mansoni*. These results are in agreement with findings by Krauth et al. [15].

The severity of disease burden at a location is generally given by two parameters, prevalence and intensity of infections. If negative individuals are included in the calculation of the mean infection intensity the high number of zeros will skew the mean downwards, especially in lower prevalence settings. Thus, a strong correlation to the prevalence will be induced, making the mean infection intensity to be a mixed measure of prevalence and infection intensity. We decided to only include positive individuals in the mean infection intensity to separate the effects of the sensitivity on observed prevalence from that on infection intensity. Therefore, we decided to estimate the mean infection intensity from only positive individuals.

It is evident from our estimates that the sensitivity of the Kato-Katz technique in hookworm is dominated by the large day-to-day variation in egg output and has only a weak dependence on infection intensity. Thus, increasing the number of samples is an effective strategy to increase sensitivity even in low transmission settings for hookworm. In contrast, for *S. mansoni* the infections that give false-negative results are largely those with light intensity. Increasing the number of samples to more than two does only marginally improve the sensitivity because the sensitivity is limited by the low density of eggs and not the variation in excretion and production. However, taking two thick smears from the same samples at 100 EPG mean infection intensity would increase the sensitivity from 50% to 70% for a comparably low additional effort. For mean infection intensities below 100 EPG no directly representative data was included in this model study. Nevertheless, extrapolations to low prevalence and infection intensity settings are valid because the model considers individual-level data. In a high intensity setting there are still many individuals with low intensity infections, and therefore our model includes information on the full range of infection intensities. Thus, inference about a low intensity and prevalence population consisting primarily of light infections is possible without making unreasonable extrapolations. For infections with an intensity above 240 EPG, the strong relation between infection intensity and sensitivity suggests that the sensitivity for two samples is close to 100%, ensuring that the most heavily infected individuals are detected and can be treated. Still, the results indicate a possibility of bias when comparing different study sites. The infection intensity-dependent sensitivity will become increasingly important in the new era with the goal to eliminate STH and schistosomiasis [38, 39].

In the study sites of Zouatta and Azaguié, the observed mean infection of *S. mansoni* was moderate compared to Fagnampleu. Hence, it is conceivable that there was a comparably larger share of light infections that were missed due to the low sensitivity of the Kato-Katz technique, and therefore, the ‘true’ mean infection intensity will be even lower. Moreover, the difference between estimated and observed prevalence is also larger due to the higher number of missed cases. For Fagnampleu, the observed mean infection intensity and therefore the overall sensitivity are high. Thus, the estimates agree with the observations and the ‘true’ prevalence is only slightly higher than the one observed. For hookworm, the likelihood of correctly diagnosing a heavy infection is still larger than for a light infection. Accordingly, the difference between the observed and the estimated mean infection intensity is larger the fewer the number of samples taken.

Our estimates of the underlying ‘true’ age-prevalence for *S. mansoni* and for hookworm are in agreement with those obtained from transmission models [19, 40] and from latent class statistical models [41]. For example, the *S. mansoni* age-prevalence is comparable to those obtained by the Yang et al. [42] models, which differentiate between the influence of water contact patterns and the acquired immunity. However, due to large uncertainty in older age groups, our results do not allow choosing the transmission model, which resembles best. For hookworm, a peak shift, as proposed by Woolhouse [43], is clearly visible in our age-prevalence curve. The decreasing prevalence at older age is apparent in all locations but it has yet to be discussed in greater detail in the literature. It could indicate a significantly lower life expectancy for infected individuals although other explanations and confounding factors are conceivable but cannot be tested with the available data.

### Conclusions

The proposed model succeeds in predicting the intensity-dependent sensitivity of the Kato-Katz technique directly from the day-to-day variation in helminth egg output. Hence, the model is able to explain the differences between the sensitivity of hookworm and *S. mansoni*. The sensitivity of Kato-Katz for hookworm is dominated by a high day-to-day variation. We recommend collecting at least two stool samples over subsequent days combined with the given sensitivity values to estimate ‘true’ prevalence. For *S. mansoni* infection the sensitivity is largely driven by light infections that are hard to detect by a single Kato-Katz thick smear. We also recommend collecting two samples due to almost perfect sensitivity for moderate and heavy infections and low benefit of additional samples for light infections. We predict that improving the sensitivity for *S. mansoni* can be achieved more cost effectively by increasing the number of Kato-Katz thick smears from the same stool sample instead of increasing the number of samples taken. Additionally, it is necessary to take into account the infection intensity-dependent sensitivity of Kato-Katz for *S. mansoni* when comparing data from several studies. Including the infection dependence becomes more important when close to elimination due to the larger changes in sensitivity of Kato-Katz with infection intensity.

A further consequence of the results is due to the fact that the guidelines of WHO are defined in terms of observed prevalence. An observed prevalence of e.g. 10% for *S. mansoni*, which is the lower limit for yearly MDA, is indicative of a ‘true’ prevalence of roughly 14%, 20%, and 29% for 200 EPG, 100 EPG, and 50 EPG, respectively. Hence, the observed prevalence is a measure of both, the ‘true’ prevalence and the infection intensity. We advise the disentanglement of these two components by defining thresholds separately for ‘true’ prevalence and infection intensity. The results also suggest that the current disease burden estimates underestimate the true prevalence.

The spline model for age-dependence used in this study can be replaced by appropriate transmission models to determine which age groups should be treated and how frequently that has to happen to increase the intervention effectiveness. The model can be further extended to analyze studies with multiple Kato-Katz thick smears performed per stool sample and thus separate day-to-day from within-sample variation. This would enable us to address the question of how repeated testing of the same sample compares to taking several samples in order to reduce cost and increase compliance.

## Supporting information

S1 Appendix A more detailed description of the study sites and data collection procedures. (PDF)

S1 Table Individual level data of the three studies sites. (XLSX)
