# Supplementary material for: Estimating sensitivity of the Kato-Katz technique for the diagnosis of Schistosoma mansoni and hookworm in relation to infection intensity
Source: PLoS Negl Trop Dis. 2017 Oct 4;11(10):e0005953. doi: 10.1371/journal.pntd.0005953 (PMC5643140; doi:10.1371/journal.pntd.0005953)
Supplement: S1 Appendix — (PDF) [file pntd.0005953.s001.pdf]

S1 data description

Zouatta. This dataset was obtained from a study carried out in Zouatta II in the district of Man, western Côte d'Ivoire in 2002, a region where *S. mansoni* is known to be endemic. Stool and urine samples were obtained from 561 individuals over three consecutive days. 545 individuals provided at least 2 stool samples with similar numbers of male and female participants. The age range was from 5 days to 91 years with a median of 17 years and a mean of 23 years. Single 41.7 mg Kato-Katz thick smears were prepared from each stool sample and examined under a light microscope. Eggs of *S. mansoni*, hookworm, *Ascaris lumbricoides*, and *Trichuris trichiura* were counted and recorded separately. 40.9% of the participants delivered at least one positive sample for *S. mansoni*, 35.3% for hookworm, 5.3% for *T. trichiura*, and 1.7% for *A. lumbricoides*.

Azaguié. The study was carried out in the two villages, Azaguié Makouguié and Azaguié M'Bromé in the district of Azaguié in south Côte d'Ivoire between June and September 2011. *S. mansoni* and *S. haematobium* are known to co-occur in this area. Stool samples were collected over two consecutive days and two Kato-Katz thick smears were prepared from each sample. 501 individuals completed the study (241 males and 260 females). The age ranged from 3 months to 82 years, with a mean age of 16.3 years, and a median age of 6 years. The overall prevalence for *S. mansoni* was 42.7%, that of hookworm was 14.2%, *T. trichiura* was 5.4%, and *A. lumbricoides* 2.4%.

Fagnampleu. Data were obtained from the initial diagnosis from a clinical trial done between November 1998 and July 1999 in the region of Man in western Côte d'Ivoire. *S. mansoni* is known to be endemic in the region and there has been no control campaign before the study. Stool samples were taken and Kato-Katz thick smears prepared and analyzed for soil-transmitted helminths and *S. mansoni* eggs over four consecutive days from 354 schoolchildren. The age range was from 6 to 15 years, the mean 9.4 years, and the median 9 years. The overall prevalence was 76.3% for *S. mansoni*, 59.0% for hookworm, 1.6% for *T. trichiura*, and 0.8% for *A. lumbricoides*.
